# Optimized Chemical Extraction Methods of Antimicrobial Peptides from Roots and Leaves of Extremophilic Plants: *Anthyllis sericea* and *Astragalus armatus* Collected from the Tunisian Desert

**DOI:** 10.3390/antibiotics11101302

**Published:** 2022-09-24

**Authors:** Raoua Ben Brahim, Hasna Ellouzi, Khaoula Fouzai, Nedra Asses, Mohammed Neffati, Jean Marc Sabatier, Philippe Bulet, Imed Regaya

**Affiliations:** 1 Laboratory of Extremophile Plants, Centre of Biotechnology of Borj Cedria, B.P. 901, Hammam-Lif 2050, Tunisia; 2 Higher Institute of Biotechnology of Monastir, University of Monastir, Monastir 5000, Tunisia; 3 Department of Biology, Faculty of Sciences of Bizerte, Carthage University, Bizerte 7021, Tunisia; 4 Higher Institute of Sciences and Technologies of the Environment of Borj Cedria, University of Carthage, Amilcar 1054, Tunisia; 5 Laboratory of Pastoral Ecosystems and Valorization of Spontaneous Plants, LR16IRA03, Institute of Arid Regions, University of Gabès, Médenine 4119, Tunisia; 6 Institut de Neurophysiopathologie (INP), Faculté des Sciences Médicales et Paramédicales, Aix-Marseille Université, CNRS UMR 7051, 27 Bd Jean Moulin, 13005 Marseille, France; 7 Plateform BioPark Archamps, 218 Avenue Marie Curie Archparc, 74160 Archamps, France; 8 CR University Grenoble Alpes, Institute for Advanced Biosciences, Inserm U1209, CNRS UMR 5309, 38700 Grenoble, France

**Keywords:** Extremophile plants, antimicrobial peptides, extraction solvent, optimization, *Anthyllis sericea*, *Astragalus armatus*

## Abstract

Extraction methods depend mainly on the chemical nature of the extracted molecule. For these reasons, the selection of the extraction medium is a vital part of obtaining these molecules. The extraction of antimicrobial peptides (AMPs) from extremophile plants is important because of its potential pharmaceutical applications. This work focused on the evaluation of several solvents for the extraction of AMPs from the following two extremophile plants: *Astragalus armatus* and *Anthyllis sericea* from southern Tunisia. In order to identify the most efficient solvents and extraction solutions, we used sulfuric acid, dichloromethane, phosphate buffer, acetic acid and sodium acetate, and we tested them on leaves and roots of both the studied plants. The extracts obtained using sulfuric acid, dichloromethane and phosphate buffer extraction did not show any antimicrobial activity, whereas the acetic acid and sodium acetate extracts led to growth inhibition of some of the tested bacterial strains. The extracts of leaves and roots of *An. sericea* and *As. armatus* obtained by acetic acid and sodium acetate were proven to be active against Gram-positive bacteria and Gram-negative bacteria. Therefore, the most appropriate solvents to use for antimicrobial peptide extraction from both plants are acetic acid and sodium acetate.

## 1. Introduction 

The fight against multidrug-resistant bacterial infections is gaining more and more attention worldwide [1,2]. Several methods have been developed, such as phagotherapy, anti-sense ARNs, nanoparticles and antimicrobial peptides (AMPs) [3]. Botanicals are among the most promising resources for research into new antibacterial agents [4].

In addition to phenols, alkaloids, and some plant secondary metabolites with known antimicrobial characteristics, AMPs from plants can be beneficial for this purpose [5,6]. AMPs play a significant role in innate immunity and are very often part of the front line of defense against invading pathogens [7]. AMPs produced by plants are very similar in structure and function to AMPs produced by humans and within the animal kingdom generally [6]. In general, AMPs are small molecules with a molecular weight of 2–10 kDa, are mainly positively charged, have amphiphilic properties, and have a basic pH value [8,9]. A variety of antimicrobial peptides have been identified over the past two decades. Today, more than 5000 antimicrobial peptides have been isolated from living organisms, 368 of which are from plants [9]. Only 3425 AMPs are listed in the antimicrobial peptide database (APD34) [10].

Plants have the ability to produce, after receiving microbial signals, various antimicrobial substances such as restriction enzymes, and an arsenal of protein molecules such as AMPs [11].

*An. sericea* and *As. armatus*, two Saharan plant species, were collected from the arid regions of Tunisia and more precisely from the Medenine region, in order to study their antimicrobial activity. These two extremophile plants were selected based on the criterion of their geographical distribution. *An. serecea* was collected in a mountainous Saharan zone and *As. armatus* was collected in a Saharan zone closer to the marine coast.

AMPs are of particular interest among all groups of substances of plant origin because they have diverse biological functions, such as antibacterial [12,13] and antifungal functions [14], and the inhibitory activity of trypsin and α-amylase [15]. Antimicrobial peptides can be extracted from various parts of plants, since they are produced in seeds, roots, stems, leaves and flowers [16]. Previous studies revealed that extremophilic plants represent a real reservoir of interesting active biomolecules that are of biological or environmental interest [17,18].

It should be mentioned that the exploitation of such plant resources has generally been restricted to the extraction of essential oils, polyphenols and flavonoids. Indeed, the extraction of natural peptides is essential because AMPs represent a very promising alternative to classic chemical antibiotics. 

*An. sericea* is a medium-sized plant with ligneous branches and greenish-gray leaves [19]. It is a wild endemic plant, belonging to the leguminous family, that is found in the south of Tunisia, in the arid region, and grows in severe environmental conditions. It is exclusively found on calcareous crusts possibly covered by a fine soil. It has a size of between 5 and 40 cm, and is recognized especially for its therapeutic properties. This spiny plant is used in popular medicine for the treatment of infections [20]. As a result of chemical analyses which were carried out, the presence of 4-methyl ether was detected in both leaves and stems of *Anthyllis sericea* [21] and their richness in flavonoids was proved [19].

*As. armatus* is a North African endemic shrub, found in near-Saharan areas. [22]. *Astragale,* which belongs to the legume family *Fabaceae* and is a member of the subfamily *Faboideae*, is a genus with about 2500 species and is considered to be the largest and most complex genus of angiosperms [23,24]. Some varieties are popularly used for chronic pain, fever, feverishness, hypertension, arthritis, scorpion stings and snake bites, stomach problems and diabetes [25,26]. Its aerial parts are rich in glycoside and flavonol, and the seeds are rich in protein, lipids, carbohydrates and minerals [25]. 

Despite their well-known advantages in traditional medicine and the research carried out on their composition, no study has been carried out to date on the AMPs of these two plants according to our searches in the databases of AMPs. For this reason, in this research, we are mainly interested in the extraction of these antimicrobial compounds from the roots and leaves of the two plants concerned. It should be noted that in previous studies of the literature on various plant species, a variety of AMP extraction methods have been developed. The development of such methods was based both on the characteristics of these plant tissues and on the specific solubility of the peptides.

In this work, the extraction of AMPs from different parts of the two selected plants (as mentioned above) is achieved by a combination of mechanical means, which leads to a cell lysis that consists of a rupture of the plasma membranes of cells (grinding, agitation, etc.) and chemical means (extraction solution) to extract the molecules of interest.

## 2. Results 

### 2.1. Extraction

Lyophilized leaf and root extracts of each species were obtained by using five solvents (acetic acid, sodium acetate, dichloromethane, phosphate buffer and sulfuric acid). One gram of dry material of each organ was used in the subsequent analysis. 

The following two criteria were fixed to evaluate the efficiency of each extraction solvent: (i) extract efficacity in the inhibition of tested microbial strains; (ii) protein content of the crude extract.

### 2.2. Antimicrobial Activity

**Figure 1:**
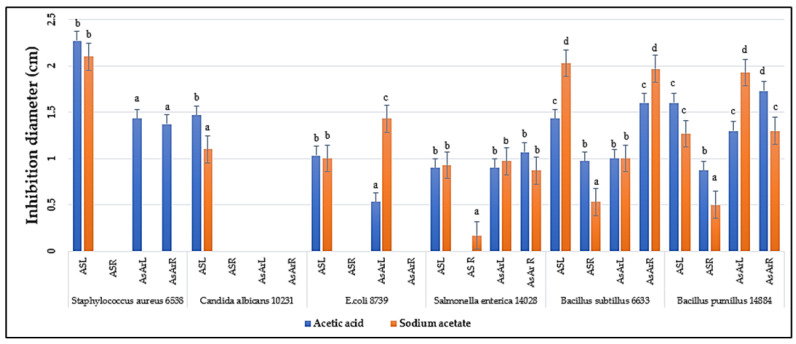
Inhibition of different strains by acetic acid and sodium acetate roots and leaves extracts. Means followed by the same or common letter (s) are not significantly different among studied solvent based on the Duncan test at 5%.

Inhibitory activities of various parts of the two plants were evaluated against six bacterial strains. Extracts from both *Anthyllis sericea* and *Astragalus armatus* obtained from the different extractions showed variable efficacy against the tested strains. The extracts derived from acetic acid and sodium acetate proved to be active against the studied bacterial strains, with a significant difference in the inhibition diameters (Figure 1). Indeed, the resulting products of the extractions based on the use of sulfuric acid, phosphate buffer and dichloromethane failed to show any activity on the different tested strains. We present here only the results of the extracts obtained from acetic acid and sodium acetate because these are the only solvents whose extracts showed microbial inhibitions.

The extract of *An. sericea* leaves derived from acetic acid and sodium acetate extraction proved to be active against Gram-positive bacteria (*Staphylococcus aureus* 6538, *Bacillus subtilis* 6633 and *B. pumillus* ATCC 14884) and against Gram-negative bacteria (*Escherichia. coli* ATCC 8739 and *Salmonella enterica* ATCC 14028) with a diameter ranging from 0.9 to 2.25 cm. The roots of the same plant are only active against *B. subtilis* ATCC 6633 and *B. pumillus* ATCC 14884 bacteria with an inhibition diameter less than 1 cm. The leaves and roots of *As. armatus* have been shown to be biologically active against the same bacterial strains: *Staphylococcus aureus* ATCC 6538, *B. subtilis* ATCC 6633, *B. pumillus* ATCC 14884 and *Salmonella enterica* ATCC 14028.

The roots and leaves of the two plants studied, *As. armatus* and *An. Serecea,* therefore contain agents with antimicrobial properties, among which the leaves of *An. sericea* are the most active.

### 2.3. Protein Content of the Extracts

**Figure 2:**
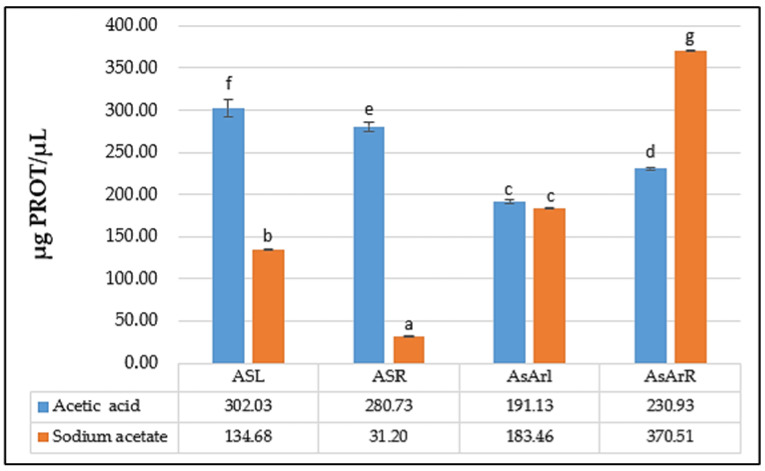
The amount of protein obtained by acetic acid extraction compared to that obtained by sodium acetate. Means followed by the same or common letter (s) are not significantly different among studied solvent based on the Duncan test at 5%.

The presence of proteins was observed in all leaf and root extracts of *An. sericea* and *As. armatus*. The amount of protein revealed significant differences among both solvents in all plant parts, except for the leaves of *As. armatus*, with the highest values noted for *As. armatus* roots under sodium acetate solvent, suggesting that the inhibitions shown in the antimicrobial tests are mainly due to proteins that have an antimicrobial effect. According to the results obtained from the determination of proteins with Bradford’s method, the extracts obtained from acetic acid are richer in proteins than the samples from sodium acetate (Figure 2). The amounts of protein obtained with acetic acid extraction vary between 191.13 µg/µL and 302.03 µg/µL, while those obtained by sodium acetate vary between 31.20 µg/µL and 370.51 µg/µL. Therefore, the extraction of antimicrobial peptides using acetic acid yields a higher amount of protein than extraction with sodium acetate.

### 2.4. Chromatographic Analysis

**Figure 3:**
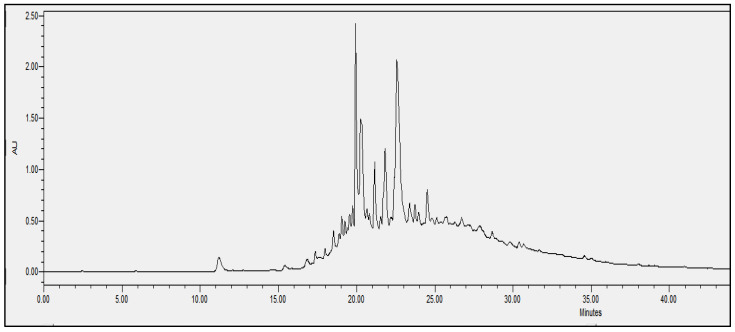
Chromatograms of *Anthyllis serecia* leaves extract obtained with acetic acid.

**Figure 4:**
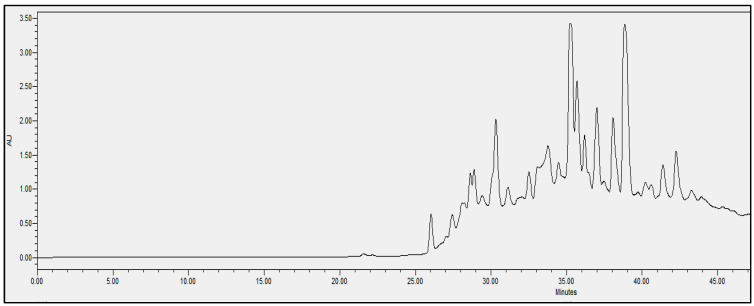
Chromatograms of *Anthyllis serecia* leaves extract obtained with sodium acetate.

**Figure 5:**
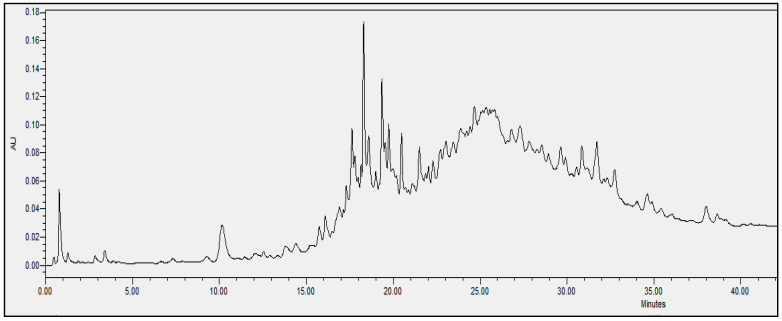
Chromatograms of *Anthyllis serecia* roots extract obtained with acetic acid.

**Figure 6:**
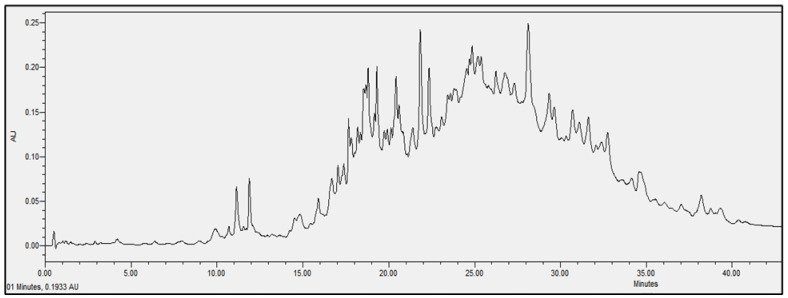
Chromatograms of *Anthyllis serecia* roots extract obtained with sodium acetate.

**Figure 7:**
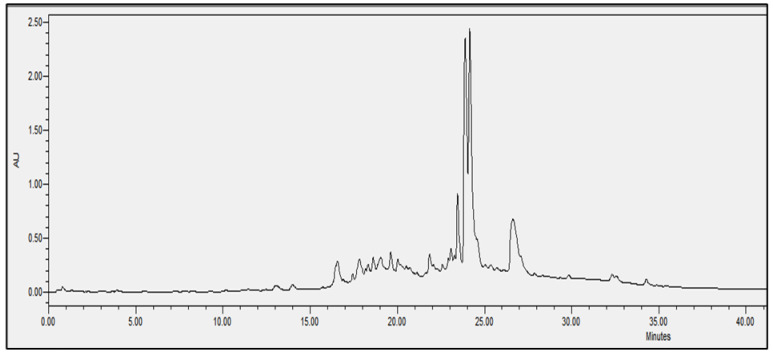
Chromatograms of *Astragallus armatus* leaves extract obtained with acetic acid.

**Figure 8:**
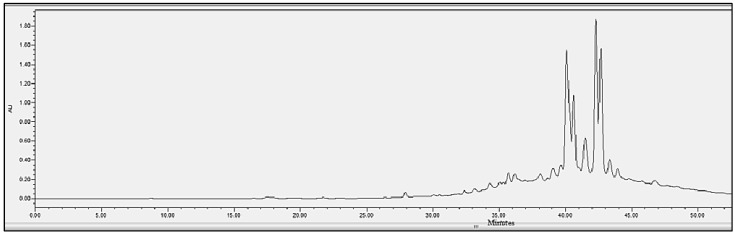
Chromatograms of *Astragallus armatus* leaves extract obtained with sodium acetate.

**Figure 9:**
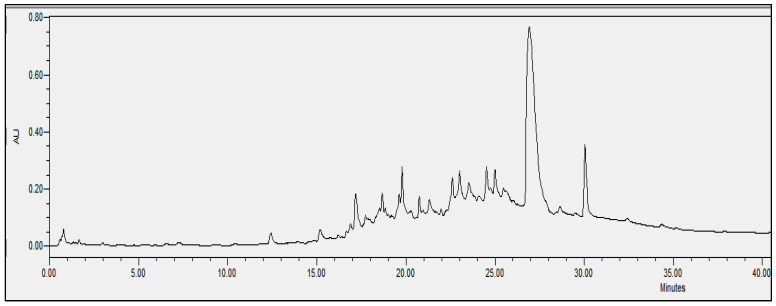
Chromatograms of *Astragallus armatus* roots extract obtained with acetic acid.

**Figure 10:**
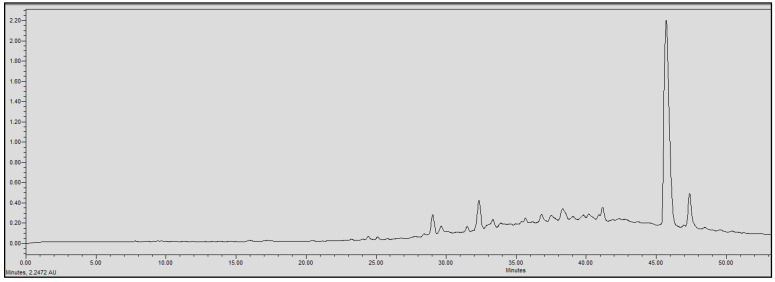
Chromatograms of *Astragallus armatus* roots extract obtained with sodium acetate.

In order to evaluate the molecular complexity of the extracts (acetic acid and sodium acetate) that showed antimicrobial activity, they were analyzed by performing UPLC. All the chromatograms obtained were detected at a wavelength of equal to 230 nm (the specific wavelength for the detection of peptide bonds of 213–230 nm). Therefore, we are certain that we obtained proteins with both extraction methods, i.e., with either acetic acid or sodium acetate solvent. The chromatographic profiles of the roots and leaves (Figure 3, Figure 4, Figure 5, Figure 6, Figure 7, Figure 8, Figure 9 and Figure 10) of the two plants are different from each other according to the extraction medium (especially in the number of peaks and hydrophobicity/hydrophilicity of the compounds), considering that each peak may contain at least one molecule.

For example, regarding the acetic acid extracts, most of the molecules are eluted at between 8% and 34% ACN, while for sodium acetate the highest number of compounds are eluted at between 20% and 40% ACN. The only exception is for the *An. sericea* roots extract, as both elution profiles are very similar despite the extraction condition and they both have a more complex profile of between 18% and 28% ACN. 

This proves molecular diversities among these plants, and even among the different parts of the same plant.

Extraction by acetic acid and sodium acetate yielded both active extracts but with different chromatographic profiles, supporting the hypothesis of an interesting molecular variety, and confirming the importance of testing several extraction procedures. 

In this study, we demonstrated that the molecular profiles are different depending on the extraction procedure, the plant tissue and also the extraction conditions. 

## 3. Discussion 

Different research works have been published on the various health purposes and uses of the entire parts of the plants or on their extracts in different solvents. Currently, researchers are interested in natural antimicrobial peptides (AMPs) since they are expected to be potent antimicrobial agents with no side effects.

Antimicrobial peptides derived from plants can be obtained in different ways, and the success of this process can be strongly affected by the choice of extraction solvent. From the earliest research on the isolation of AMPs from plants, the extraction of these molecules was based on the use of aqueous saline solutions, dilute acids or buffers [27]. The purothionines, which, as already mentioned, are the first AMPs obtained from plants, were extracted with a sulfuric acid solution [28,29]. The extraction of AMPs from plant sources is currently achieved by using either water or water-based solutions, such as acids, buffers and salt solutions, or by using organic solutions. Masatoshi Fujimura successfully extracted and identified two AMPs, Fa-AMP1 and Fa-AMP2, from the seeds of buckwheat (*Fagopyrum esculentum* Moench) by employing sodium acetate as an extraction solvent [12]. 

In this study, the extraction of AMPs from *As. armatus* and *An. serecia* roots and leaves was carried out for the first time. The classification of AMPs is based on their composition in amino acids, and consequently on their molecular charge and their secondary structures. Most plant AMPs, despite their molecular diversity, share common characteristics, in that they are basic, amphipathic, cysteine-rich peptides with disulphide bond-stabilized structures. 

Generally, AMPs contain hydrophobic residues, such as alanine, leucine, phenylalanine and tryptophan. The majority of AMPs are positively charged at a physiological pH due to the strong presence of basic amino acid residues such as lysine and arginine, although some anionic AMPs are also known [30]. Therefore, such diversity in structure and chemical characteristics requires different extraction solvents with a varying polarity, pH and degree of hydrophobicity in order to extract the different types of AMPs. Since the extraction of AMPs from the two plants studied had not been carried out before, we investigated different reagents and solvents that were successful in obtaining AMPs from other plants in the current study, so that we could identify the most suitable solvent for AMP extraction from *As. armatus* and *An. sericea*. 

In the present study, the extraction of AMPs from biomass in an organic acid (acetic acid) and its conjugate coupled with a sodium (sodium acetate) proved to be more efficient; the chemical structure of the solvent or buffer favored or opposed the solubilization of any particular molecule from a specific system. Although dichloromethane, phosphate buffer and sulfuric acid have been used successfully with other plant tissues, they were not found to be effective in this case. The antimicrobial tests of the different extracts obtained demonstrated that an interesting antimicrobial activity is obtained using an acetic acid or sodium acetate extractant. 

As summarized in Figure 1, the leaves and roots of *As. armatus* from acetic acid and sodium acetate extractions were biologically active against the same bacterial strains, namely *Staphylococcus aureus* ATCC 6538, *Bacillus subtilis* ATCC 6633, *B. pumillus* ATCC 14884 and *Salmonella enterica* ATCC 14028. The extract of leaves of *An. sericea* derived from the extraction with acetic acid and sodium acetate was found to be active against Gram+ bacteria, i.e., *Staphylococcus aureus* ATCC 6538, *B. subtilis* ATCC 6633 and *B. pumillus* ATCC 14884, and against Gram- bacteria, i.e., *E. coli* ATCC 8739 and *Salmonella enterica* ATCC 14028, whereas the roots of the same plant are only active against *B. subtilis* ATCC 6633 and *B. pumillus* ATCC 14884. 

Therefore, these results demonstrate that acetic acid and sodium acetate were used to extract AMPs from the roots and leaves of the two studied plants.

The protein assays and the chromatographic profiles proved the presence of proteins. 

According to the results obtained from the determination of proteins with Bradford’s method (Figure 2), all the extracts obtained from acetic acid and acetate sodium from either leaves or roots of *An. sericea* and *As. armatus* revealed the presence of proteins. According to the same results, the extracts obtained from acetic acid are richer in proteins than the samples from sodium acetate. Therefore, the extraction of antimicrobial peptides using acetic acid makes it possible to obtain a higher protein amount than extraction with sodium acetate.

The chromatographic results confirmed the results of the protein assays. The profiles of the roots and leaves (Figure 3, Figure 4, Figure 5, Figure 6, Figure 7, Figure 8, Figure 9 and Figure 10) of the two plants are different from each other, and even the general profiles of the same organ are different from one medium to another. For example, peak retention time, width of peaks, and intensity of peaks are different under the same gradient and the same conditions of injection. We consider that the base width of each peak is proportional to the molecular weight of the molecule (the larger the peak, the higher the molecular weight). Even if the peaks are similar in width and height but different in elution, we estimate that the physicochemical properties of the extracted compounds are different. From this, we can conclude that our extracts contain an interesting variety of proteins to be separated and identified.

The whole crude extract obtained was analyzed by performing UPLC (to obtain the crude profile) at 213–230 nm, which is the wavelength of the peptide bonds, and all the peaks were collected together to avoid the loss of the initial crude extract injected. The collected set of crude peaks (detected at 214–230 nm) was lyophilized and tested for antimicrobial activity and was found to have identical results to the starting crude extract, which suggests that the inhibitions witnessed in the antimicrobial assays derive essentially from proteins that have antimicrobial properties. In our work, we were able to verify the presence of AMPs as bioactive molecules responsible for antibacterial activity based on the following set of bibliographic parameters and biochemical indicators confirming their presence: (i) the choice of AMP precipitation solvents already mentioned in the literature, (ii) the detection of chromatographic profiles at a specific wavelength of the peptide bonds, and (iii) the Bradford test that was performed afterwards to determine the total peptides obtained (the same sample was analyzed by performing UPLC and was tested for its biological activity).

*As. armatus* and *An. serecia* have shown inhibitory activity against pathogenic and antibiotic resistant strains, which provides incentive to further study other parts such as the seeds and even work towards the fractionation, purification and identification of AMPs present in these plants. The adaptation of these species to extreme environments has necessarily engendered the development of new biosynthetic pathways, giving rise to new molecules that could be applied in the treatment of various diseases.

## 4. Material and Methods

### 4.1. Plant Material 

*An. sericea* and *As. armatus* plants were collected from the arid regions of the Tunisian desert and more precisely from the Medenine region.

### 4.2. Bacterial Strains

*Salmonella enterica* ATCC 14028, *Escherichia coli* ATCC 8739, *Bacillus subtilis* ATCC 6633, *Bacillus pumilus* ATCC 14884, *Staphylococcus aureus* ATCC 6538 and *Candida albicans* ATCC 10231 are the microorganisms that were used to test the inhibitory activity of the plant extracts; they were provided by the laboratory of biotechnology of CBBC, Borj Cedria Tunisia. These strains were especially selected for their pathogenicity as they cause hospital infections and antibiotic resistance [31,32,33,34,35].

### 4.3. Extraction of Antimicrobial Peptides

For each plant, 1 g of the leaves and roots was weighed and used for extraction as per the following five methods. All extraction steps were realized overnight under shaking at 4 °C. 

The first extraction was based on the work of Zhang and Lewis in 1997 [36]. The plant material (roots, leaves) of both plants was mixed with 30 mL of 50 mM sulfuric acid solution. The second extraction refers to the work of Fujimura et al., 2003 [12], whereby the plant materials (1 g of each part) were homogenized in 30 mL of 50 mM sodium acetate. The third extraction was performed by homogenizing the plant materials in 30 mL of dichloromethane, according to the work performed by Menonvé Atindehou in 2013 [37]. Based on the work of Song et al. published in 2004, the fourth extraction of the plant materials was achieved by homogenization with 30 mL of the 20 mM phosphate buffer at pH 7.2 containing 0.1 M sodium chloride; the pH of the extracts was adjusted to 4 with acetic acid [38]. Finally, the fifth extraction was performed in a solution of 1 M acetic acid.

**Figure 11:**
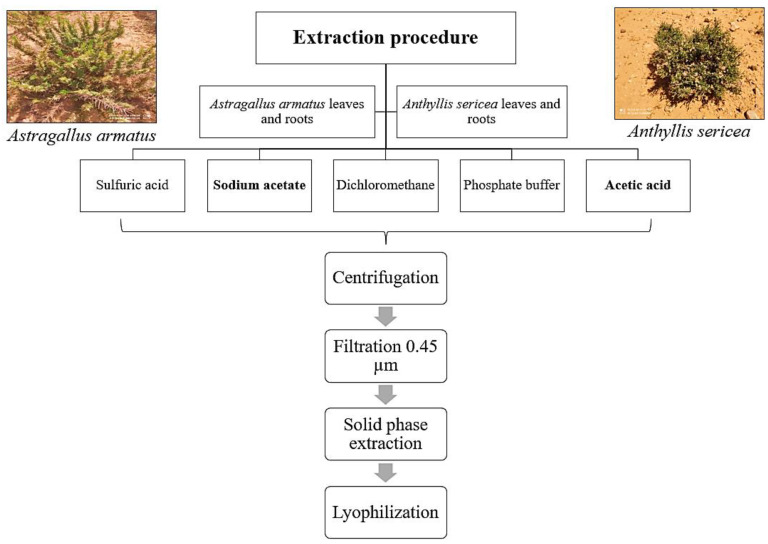
Summary of extraction procedure.

After incubation, each extract was centrifuged at 9000 rpm for 30 min. The supernatant was filtered through a 0.45 µm syringe filter and the filtrate was subjected to solid-phase extraction (C_18_ cartridge). The eluate obtained (60% acetonitrile in acidified water, 0.1% TFA) was lyophilized and the dried eluates obtained were kept at −20 °C until use. The process of extraction is briefly summarized in Figure 11.

### 4.4. Antimicrobial Activity Tests

A pre-culture of each strain was performed on plate count agar (PCA) medium and then incubated overnight at 37 °C to obtain an exponential growth phase. For each strain, the optical density was measured at 600 nm and adjusted to obtain a bacterial suspension of approximately 1000 bacteria per mL. PCA plates were then inoculated with this culture and wells were dug with the yellow cones. A total of 20 µL of plant extract was applied in each well. After 24 h of incubation at 37 °C, the zone of growth inhibition was measured [39,40]. The test was carried in triplicate. 

### 4.5. Protein Quantification 

Soluble proteins were quantified according to the Bradford method. Aliquots of 100 µL of each extract were mixed with 200 µL of Bradford reagent and 700 µL of distilled water. The staining developed in 15 min of dark incubation. Then, the absorbance was read at 595 nm, with reference to a blank sample without protein extract. The soluble protein content was determined by referencing a bovine serum albumin (BSA) standard range [41]. 

### 4.6. Liquid Chromatography (UPLC) 

The extracts were purified by performing ultra-performance liquid chromatography (UPLC, Acquity) with a UV detector and Empower V3 software (Waters, Milford, MA). The mobile phase was composed of solvent A (H_2_O 0.1% TFA) and B (Acetonitrile 0.1% TFA), with a gradient of 2 to 60% acetonitrile at a flow rate of 0.3 mL/min for 70 min. The used column is an analytical HPLC C18 column (4.6 × 250 mm) and the detection was carried out at a wavelength of 230 nm. 

### 4.7. Statistical Analysis

Statistical analysis was carried out using the software package SPSS version 21.0 (SPSS Inc., Chicago, IL, USA). Differences between different species at a given solvent type were determined by a One-Way ANOVA according to Duncan’s multiple range tests (*p* ≤ 0.05).

## 5. Conclusions

The objective of the research, which was carried out with the extraction of antimicrobial peptides from two extremophilic plants, was to evaluate the plants’ activity using different extraction solvents. The results obtained demonstrate that the lyophilized extracts obtained from the acetic acid and sodium acetate extraction have an inhibitory effect on both plants for Gram+ bacteria (*Staphylococcus aureus*, *Bacillus subtilis*, *B. pumillus*) and Gram- bacteria (*E. coli*, *Salmonella enterica*). The protein assay carried out made it possible to determine the amount of protein contained in the different extracts. Moreover, on the basis of the chromatographic analysis carried out by performing UPLC, we deduced that the extracts of our two plants were, interestingly, rich in peptides. Finally, it is worth mentioning that the present study is the first to investigate AMPs in both *As. armatus* and *An. Sericea*, from southern Tunisia. The antimicrobial activity of these two species has been demonstrated. This study will be supplemented by the purification, characterization and identification of the molecules of each extract involved in their antimicrobial activity.
